# Recent Advances in the Treatment of Sickle Cell Disease

**DOI:** 10.3389/fphys.2020.00435

**Published:** 2020-05-20

**Authors:** Gabriel Salinas Cisneros, Swee L. Thein

**Affiliations:** ^1^Sickle Cell Branch, National Heart Lung and Blood Institute, National Institutes of Health, Bethesda, MD, United States; ^2^Division of Hematology and Oncology, Children’s National Medical Center, Washington, DC, United States

**Keywords:** sickle cell disease, anti-sickling agents, gene editing, gene therapy, hemoglobinopathies

## Abstract

Sickle cell anemia (SCA) was first described in the Western literature more than 100 years ago. Elucidation of its molecular basis prompted numerous biochemical and genetic studies that have contributed to a better understanding of its pathophysiology. Unfortunately, the translation of such knowledge into developing treatments has been disproportionately slow and elusive. In the last 10 years, discovery of *BCL11A*, a major γ-globin gene repressor, has led to a better understanding of the switch from fetal to adult hemoglobin and a resurgence of efforts on exploring pharmacological and genetic/genomic approaches for reactivating fetal hemoglobin as possible therapeutic options. Alongside therapeutic reactivation of fetal hemoglobin, further understanding of stem cell transplantation and mixed chimerism as well as gene editing, and genomics have yielded very encouraging outcomes. Other advances have contributed to the FDA approval of three new medications in 2017 and 2019 for management of sickle cell disease, with several other drugs currently under development. In this review, we will focus on the most important advances in the last decade.

## Introduction

Sickle cell disease (SCD) is an inherited blood disorder that first appeared in the Western literature in 1910 when Dr. James Herrick described a case of severe malaise and anemia in a 20-year-old dental student from Grenada (Herrick, 1910). On examining his blood smear, he noticed many bizarrely shaped red blood cells, leading him to surmise that “…the cause of the disease may be some unrecognized change in the red corpuscle itself” (Herrick, 2014). More than 100 years later we recognize that the change in the red corpuscle is caused by a single base substitution in β-globin, and that the disease is not just present in the United States (US), but prevalent in regions where malaria was historically endemic, including sub-Saharan Africa, India, the Middle East, and the Mediterranean (Williams and Thein, 2018). Presence of SCD in the non-malarial regions is related to the recent migration patterns.

Currently, an estimated 300,000 affected babies are born each year, more than 80% of whom are in Africa. Due to recent population migrations, increasing numbers of individuals affected by SCD are encountered in countries that are not historically endemic for malaria, such as the US. It is estimated that 100,000 Americans are affected with SCD, the majority of whom are of African descent (Hassell, 2010, 2016). The numbers affected with SCD are predicted to increase exponentially; Piel et al. (2013) estimated that between 2010 and 2050, the overall number of births affected by SCD will be 14,242,000; human migration and further globalization will continue to expand SCD throughout the world in the coming decades. While 75% or more of newborns with SCD in sub-Saharan Africa do not make their fifth birthday (McGann, 2014), in medium- to well-resourced countries almost all of affected babies can now expect to live to adulthood but overall survival still lags behind that of a non-SCD person by 20–30 years (Telfer et al., 2007; Quinn et al., 2010; Elmariah et al., 2014; Gardner et al., 2016; Serjeant et al., 2018). Despite these global prevalence figures, and the fact that SCD is by far the largest public health concern among the hemoglobinopathies, it was not until 2006 when the World Health Organization (WHO) recognized SCD as a global public health problem^1^.

In 1949, Linus Pauling showed that an abnormal protein (hemoglobin S, HbS) was the cause of sickle cell anemia (SCA), making SCD the first molecular disease and motivating an enormous amount of scientific and medical research. Because of its genetic simplicity, SCA has been used to illustrate many of the advances in molecular genetics such as detection of a DNA mutation by restriction fragment enzyme analysis, and was used as proof of principle for the polymerase chain reaction (PCR) that we now take for granted (Wilson et al., 1982; Saiki et al., 1985).

In the last 50 years, tremendous progress has been made in understanding the pathophysiology and pathobiological complexities of SCD, but developing treatments has been disproportionately slow and elusive; a history of Perils and Progress, so succinctly summarized by Wailoo (2017). We are confident that in the next 30 years, the therapeutic landscape for SCD will change due to a combination of recent advancements in genetics and genomics, an increase in the number of competing clinical trials, and also an increased awareness from the funding bodies, in particular the NIH, USA.

**FIGURE 1:**
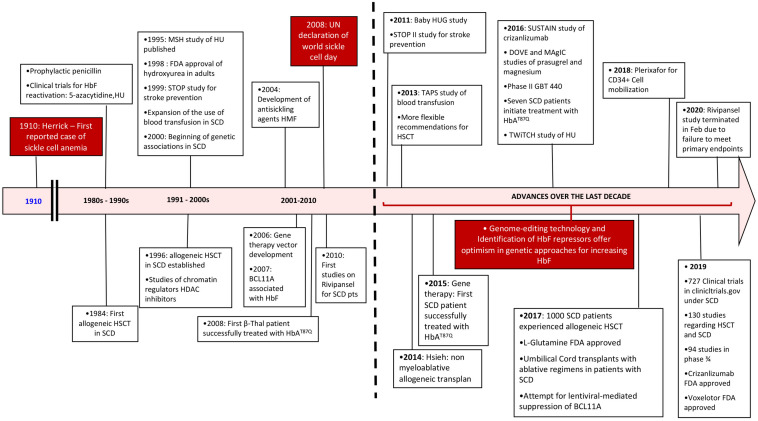
Timeline review of historic events since the diagnosis of sickle cell disease with an emphasis over the last decade. SCD, sickle cell disease; HSCT, hematopoietic stem cell transplant; HU, hydroxyurea.

Here, after a brief review of the pathophysiology, we will focus on the advances in treatment of SCD that have occurred in the last 10 years and that have reached phase 2/3 of clinical trials (Figure 1).

## Pathophysiology of Sickle Cell Disease

Sickle cell disease is caused by an abnormal HbS (α_2_β^S^_2_) in which glutamic acid at position 6 of the β-globin chain of hemoglobin is changed to valine. Goldstein et al. (1963) showed that this amino acid substitution arose from a single base change (A>T) at codon 6 (*rs334*). The genetic causes of SCD include homozygosity for the *rs334* mutation (HbSS, commonly referred as SCA) and compound heterozygosity between *rs334* and mutations that lead to either other structural variants of β-globin (such as HbC, causing HbSC) or reduced levels of β-globin production as in β-thalassemia (causing HbS/β-thalassemia). In patients of African ancestry, HbSS is the most common cause of SCD (65–70%), followed by HbSC (about 30%), with HbS/β-thalassemia being responsible for most of the rest (Steinberg et al., 2001). SCA in which the intracellular concentration of HbS is almost 100%, is by far the most severe and well described (Brittenham et al., 1985). The majority of the therapeutic developments and interventions have focused on this genotype, which is also the focus of this review, although they also impact the other SCD genotypes.

**FIGURE 2:**
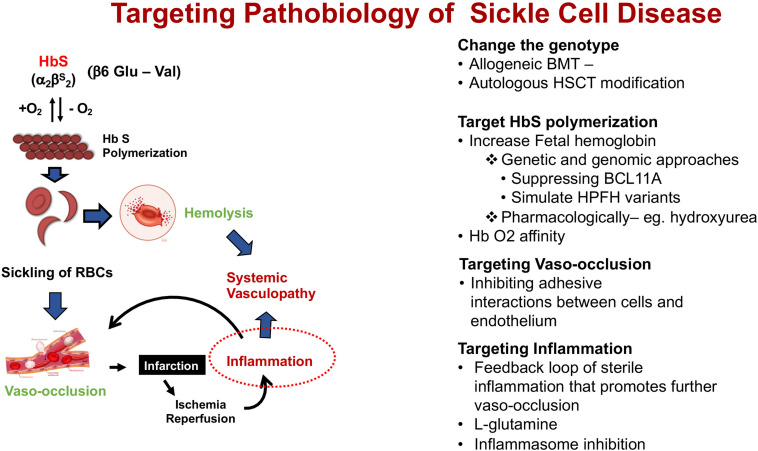
Schematic pathophysiology review of sickle cell disease and its main different targets for intervention. Hb S, hemoglobin S.

The fundamental event that underlies the complex pathophysiology and multi-systemic consequences of SCD is the polymerization of HbS that occurs under low oxygen tension (Figure 2). Polymerization of the de-oxygenated HbS alters the structure and function of the red blood cells (RBCs). These damaged (typically sickled shaped) RBCs are not only less flexible compared to normal RBCs, but also highly adhesive. Repeated cycles of sickling and unsickling shortens the lifespan of the damaged sickle RBCs to about 1/6th that of normal RBCs (Bunn, 1997; Hebbel, 2011). The outcome is the occlusion of blood vessels in almost every organ of the body and chronic hemolytic anemia, the two hallmarks of the disease, that result in recurrent episodic acute clinical events, of which acute pain is the most common, and accumulative organ damage. Acute sickle pain is so severe that it is often referred to as “vaso-occlusive sickle crisis” or VOC.

These events trigger a cascade of pro-inflammatory activity setting off multiple pathophysiological factors that also involve neutrophils, platelets, and vascular endothelium (Sundd et al., 2019). The continual release of cell-free hemoglobin from hemolysis depletes hemopexin and haptoglobin, a consequence of which is the reduced bioavailability of nitric oxide (NO), and vascular endothelial dysfunction that underlies the chronic organ damage in SCD pathology.

The sickle red blood cells do not just interact with the vascular endothelium but trigger activation of neutrophils, monocytes and platelets. During steady-state, patients with SCD have above normal values of neutrophils, monocytes and platelets which further increase during acute events (Villagra et al., 2007). Neutrophilia has been consistently correlated with SCD severity (Ohene-Frempong et al., 1998; Miller et al., 2000); neutrophils play a central role in vaso-occlusion through their interactions with both erythrocytes and endothelium upregulating expression of cytoadhesion molecules such as P- and E-selectins, current therapeutic targets (Zhang et al., 2016).

Platelets, when activated, form aggregates with erythrocytes, monocytes, and neutrophils both in patients and in murine models (Wun et al., 1997; Zhang et al., 2016). As with neutrophils, it appears that platelet aggregation is dependent on P-selectin. As part of this constant inflammatory state, the coagulation cascade is also hyperactivated in SCD. The repeated interaction between RBCs and endothelium promote expression of pro-adhesive and procoagulant proteins evidenced by increased levels of plasma coagulation factors, tissue factor (TF) and interactions between monocyte-endothelium, platelet-neutrophil and platelet-RBC. Patients with SCD have increased rates of venous and arterial thrombotic events (Brunson et al., 2017).

Unraveling these pathophysiological targets has provided insights on clinical trials on anti-platelet and anti-adhesion agents, as well as anti-coagulation factors for the prevention of acute VOC pain in SCD (Telen, 2016; Nasimuzzaman and Malik, 2019; Telen et al., 2019). A case in point is the development of an anti-P-selection molecule (Crizanlizumab) for treatment of sickle VOC, recently approved by the FDA in November 2019 and marketed as Adakveo^®^.

New therapeutic approaches that use drugs to ameliorate the downstream sequelae of HbS polymerization have not proved to be as effective as hydroxyurea (HU) which has an “anti-sickling” effect via induction of fetal hemoglobin (HbF, α2γ2) (Ware and Aygun, 2009). Other effects of HU include improvement of RBC hydration, reduction of neutrophil count, reduction of leucocyte adhesion, and reduction of pro-inflammatory markers, all of which add to the clinical efficacy of HU. In addition, HU also acts as NO donor, promoting vasodilation (Cokic et al., 2003). Increasing HbF is highly effective because it dilutes the intracellular HbS concentration, thereby increasing the delay time to HbS polymerization (Eaton and Bunn, 2017); in addition to which, the γ-chains also have an inhibitory effect on the polymerization process. Hydroxyurea, however, is only partially successful because the increase in fetal hemoglobin is uneven and not present in all cells. Nonetheless, the well-established clinical efficacy of HbF increase, substantiated by numerous clinical and epidemiological studies, has motivated both pharmacological and genetic approaches to induce HbF (Nevitt et al., 2017).

A more detailed understanding of the switch from fetal to adult hemoglobin, and identification of transcriptional regulators such as BCL11A, aided by the developments in genetic and genomic platforms, provide hope that genomic-based approaches for therapeutic reactivation of HbF may soon be possible (Vinjamur et al., 2018). In the meanwhile, a gene addition approach that infects the patient’s stem cells with a virus expressing an anti-sickling β-globin variant, T87Q, shows great promise (Negre et al., 2016; Ribeil et al., 2017). The most successful “curative” approach so far, is transplantation with stem cells from an immunologically matched sibling but this is severely limited by the lack of availability of matched donors (Walters et al., 1996a; Gluckman et al., 2017).

Parallel to the new medications being developed blood transfusions with normal red blood cells, remain an effective and increasing therapeutic option for managing and preventing SCD complications, but this strategy has limitations (not uniformly accessible, accompanied by risks of alloimmunization, hemolytic transfusion reactions and transfusional iron overload). Blood transfusion improves the oxygen-carrying capacity and improves microvascular perfusion by decreasing the HbS percentage. A major complication of blood transfusion is hemolytic transfusion reactions that occur primarily in RBC alloimmunized patients and SCD patients, in particular, are at high risk because of the mismatch in donor pool (predominantly Northern European descent) while SCD patients are predominantly of African descent (Vichinsky et al., 1990; Thein et al., 2020). Limiting blood from ethnic-matched donors has reduced but did not eliminate alloimmunization (Chou et al., 2013), and a major cause is the mismatch between serologic Rh phenotype and *RHD* or *RHCE* genotype due to variant *RH* alleles in a large proportion of the individuals (Chou et al., 2013). *RH* genotyping in addition to serologic typing may be required to identify the most compatible RBCs and recent studies have shown that a prospective rather than reactive (after appearance of allo-antibodies) genotyping approach may be feasible (Chou et al., 2018, 2020; Hendrickson and Tormey, 2018). Until prospective genotyping of RBC antigens become a practical feasibility, as a prevention, many blood transfusion centers have adopted extended red cell phenotyping, including ABO, Rh, Kell, Kidd, Duffy, and S and s antigens, and some centers have also adopted molecular genotyping for red blood cell phenotype prediction using microarray chips (e.g., the PreciseType HEA BeadChip assay). It should be noted that, while blood transfusion remains an important therapeutic option in SCD, evidence for its role in management of acute or chronic complications is lacking except for prevention of primary and secondary strokes (Howard, 2016). Supportive evidence for the role of preoperative transfusion in patients with HbSS or HbS/β^0^-thalassemia was demonstrated in the Transfusion Alternatives Preoperatively in Sickle Cell disease (TAPS) study (Howard et al., 2013).

Insight on the pathophysiology of SCD (Figure 2) has allowed different targets for interventions in patients with SCD summarized under four categories of its pathobiology – (1). Modifying the genotype, (2). Targeting HbS polymerization, (3). Targeting vasocclusion, and (4). Targeting inflammation.

Understanding of the kinetics of HbS polymerization suggest that there are many ways to inhibit HbS polymerization (Eaton and Bunn, 2017) other than induction of HbF (Table 1). One approach is to increase oxygen affinity of the hemoglobin molecule, an example is Oxbryta^TM^ (Voxelotor/GBT440) (Vichinsky et al., 2019) that was recently approved by the FDA in November 2019, making this the second anti-sickling agent.

**TABLE 1:** Current advances on therapy for sickle cell disease.

Changing the genotype		
(1) Allogeneic stem cell transplant	Myeloablative regimens (MAC), reduced intensity regimens (RIC), and non-myeloablative regimens (NMA)	50 clinical trials listed in ClinicalTrials.gov
(2) Autologous transplant		10 clinical trials listed in ClinicalTrials.gov
	a) Gene therapy	
	Lentiviral strategies (NCT02247843, NCT02140554, NCT02186418)	
	Inducing fetal hemoglobin	Downregulation of *BCL11*A (NCT03282656) Globin chromatin structure manipulation Downregulating beta^*s*^ globin expression
	b) Gene editing	
	Using zinc finger nucleosomes (ZFN), transcription activator-like effector nucleases (TALENs), CRISPR/Cas9 techniques (NCT03745287)	Downregulation of *BCL11*A Reactivation of HbF by HPFH mutations Globin gene repair
**Hemoglobin S polymerization**	Hydroxyurea (FDA approved)	Ribonucleotide diphosphate reductase inhibitor
	LBH589/Panobinostat (NCT01245179)	Pan histone deacetylase inhibitor
	Voxelotor/GBT440 (NCT03036813) (FDA approved)	α-Globin reversible binding
	Decitabine/THU (NCT01685515)	DNMT1 inhibition
	Sanguinate (NCT02411708)	Targeting carbon monoxide delivery
	IMR-687 (NCT04053803)	Phosphodiesterase 9 inhibitor
**Vasocclusion**	L-Glutamine (FDA approved)	Increase NADH and NAD redox potential
	Crizanlizumab (NCT03264989) (FDA approved)	P-selectin inhibitor
	Heparinoids: Sevuparin (NCT02515838)	P-selectin and L-selectin inhibitor
	Poloxamer and Vepoloxamer	Nonionic block copolymer surfactant
**Inflammation**	Prasugrel, ticagrelor (NCT02482298)	P2Y2 inhibitors
	Intravenous immunoglobulin (NCT01783691)	Effects on neutrophils and monocytes activation
	Simvastatin (NCT03599609)	Vascular endothelium
	Rivaroxaban (NCT02072668)	Anti factor Xa
	*N*-Acetylcysteine (NCT01800526)	Oxidative stress reduction

*HbF, hemoglobin F; HPFH, hereditary persistence of fetal hemoglobin; THU, tetrahydrouridine; DNMT1, DNA methyltransferase type 1.*

One of the biggest challenges in managing SCD is the clinical complexity and extreme variable clinical course that cannot be explained by the specific disease genotype. Patients with identical sickle genotype still display extreme clinical course; both acquired and inherited factors contribute to this clinical complexity of SCD (Gardner and Thein, 2016). Although laboratory prognostic factors (HbF, hemoglobin, reticulocyte count, leukocytosis) and clinical phenotypes (such as stroke/TIA, acute chest syndrome/pulmonary hypertension, avascular necrosis, kidney injury, or skin ulcers) have been described and analyzed, classifying disease severity remains complex and should be assessed individually. Prediction of disease severity and clinical course of SCD has been the topic of many reviews and, to date there is no clear algorithm using genetic and/or imaging, and/or laboratory markers that can reliably predict mortality risk in SCD (Quinn, 2016).

## Current Advances in Therapy

### (1) Modifying the Patient’s Genotype

Modifying the patient’s genotype via hemopoietic stem cell transplantation (HSCT) was first reported to be performed over 30 years ago in an 8-year-old child who had SCD (HbSS) with frequent VOCs; she subsequently developed acute myeloid leukemia. The patient received HSCT for the acute myeloid leukemia from an HLA-matched sister who was a carrier for HbS (HbAS). She was cured of her leukemia and at the same time, her sickle cell complications also resolved (Johnson et al., 1984; Johnson, 1985). Until then, HSCT had not been considered as a therapeutic option for SCD. This successful HSCT demonstrated that reversal of SCD could be achieved without complete reversal of the hematological phenotype to HbAA, and paved the way for bone marrow transplant (BMT) as a curative option for children with severe SCD (Walters et al., 1996b).

The conclusion was that, as long as stable mixed hemopoietic chimerism after BMT can be achieved, patients can be cured of their SCD without complete replacement of their bone marrow (Walters et al., 2001).

#### Allogeneic Bone Marrow Transplant

Hematopoietic stem cell transplant (HSCT) has now become an important therapeutic option for patients with SCD. Currently there are about 35 clinical trials at ClinicalTrials.gov studying allogeneic BMT in patients with SCD. As described by Walters et al. (2010), HSCT can establish donor-derived erythropoiesis, but even more importantly, can stabilize or even restore function in affected organs of patients with SCD when performed in time.

Between 1986 and 2013, 1,000 patients received HLA-identical matched sibling donor (MSD) HSCTs (Gluckman et al., 2017). The outcomes for both children and adults were excellent, demonstrating 93% overall survival. Eighty seven percent of the patients received myeloablative chemotherapy (MAC) and the rest (13%) received reduced intensity chemotherapy (RIC). It is important to note that patients 16 years or older had worse overall survival (95% vs. 81% *p* = 0.001) and a higher probability of graft versus host disease (GVHD)-free survival (77% vs. 86% *p* = 0.001). These results should encourage physicians to provide early referrals to SCD patients for transplant evaluation so that the donor search can be started in a timely matter (Gluckman et al., 2017).

Although myeloablative conditioning has achieved high rates of overall and event free survival, the conditioning is too toxic for adult patients with pre-existing organ dysfunction. Reversal of the sickle hematology without complete replacement of the patient’s bone marrow led to the development of less intense conditioning regimens expanding allogeneic transplantation in adult patients, who otherwise would not be able to tolerate the intense myeloablative conditioning. Donors could be HbAA or HbAS, and in order to reverse the sickle hematological genotype, the myeloid donor chimerism has to be >20% (Fitzhugh et al., 2017).

In an international, multicenter study, 59 patients had MSD HSCT, of which 50 survived and were cured of SCD. Of the nine patients that had a negative outcome, five had graft rejection and four intracranial hemorrhage. Thirteen patients developed mixed chimerism. Of those patients that developed mixed chimerism, there was no GVHD or disease recurrence/graft rejection. Patients with stable mixed chimerism did not have worse outcomes related to complications of SCD. Hsieh et al. (2009) developed a protocol for non-myeloablative HSCT with low dose total body radiation, alemtuzumab, and sirolimus. In the initial 10 patients with SCD, nine had long-term, stable, mixed donor chimerism and reversal of their sickle cell phenotype (Hsieh et al., 2009). An updated report showed that 87% of the 30 patients had long-term stable donor engraftment without acute or chronic graft-versus-host disease (Clinical trials [NCT00061568]) (Walters et al., 2001; Hsieh et al., 2014). More recent data reported at least 95% cure rate in 234 children and young adults (<30 years) with SCA after MSD with no increased mortality compared to SCA itself and better quality of life. The data also showed that myeloablative HSCT can be a safe option for patients <15 years old if a MSD is available unless there is a clear and strong recommendation not to undergo transplant (Bernaudin et al., 2020).

However, in the US, less than 15% of patients with SCD have HLA- matched siblings as donors, but a promising alternative donor source is haplo-identical family members. Studies are now underway in several centers to find a balance of conditioning regime that provides adequate immunosuppression without rejection and minimal GVHD (Joseph et al., 2018). Matched unrelated donors (MUD) have shown promising results in patients with thalassemia major and are currently being evaluated in patients with SCD (Fitzhugh et al., 2014). One of the main limitations, unfortunately, is the low probability of finding suitable donors for African and African American populations as per the National Marrow Donor Program and so, not sufficient MUD transplants have been completed in patients with SCD. HLA-haploidentical HSCT following RIC has been reported to show promising results with prolonged and stable engraftment, but for both unrelated umbilical cord blood (UCB) and haploidentical HSCT, rejection remains a major obstacle in the context of RIC (Bolanos-Meade et al., 2012; Angelucci et al., 2014; Fitzhugh et al., 2014; Saraf et al., 2018; Bolanos-Meade et al., 2019).

Although encouraging options with promising results in clinical trials, acute and chronic GVHD remain major complications which can be life threatening and have severe effects on quality of life. Multiple factors affect the development of GVHD in patients undergoing transplant, including the source of the stem cells, the intensity of immunosuppression in the conditioning regime (dose of anti-thymoglobulin) and the mismatch status of the donor to the recipient (Shenoy, 2013; Inamoto et al., 2016; Bernaudin et al., 2020).

Acute GVHD remains a concern in patients receiving mismatched donor transplants but UCB continues to show reduced rates of chronic GVHD (Kamani et al., 2012). Reduced-intensity conditioning regimens have also been studied in related and unrelated HSCT, and while a suitable option for patients with a matched sibling, patients with unrelated donor should be made aware of the not-so-favorable short and long-term outcomes (Guilcher et al., 2018).

As new transplant modalities emerge with less transplant related mortality, better immunomodulators to prevent GVHD are being developed and graft rejection has become less frequent and accepted indications for HSCT have become less restrictive (Table 2). Nonetheless, clinicians continue to have reservation toward transplant and tend to delay the referral to a HSCT specialist because of concerns for GVHD, mortality/morbidity related to transplant itself and the risk of graft rejection, which has not been eliminated completely (Leonard and Tisdale, 2018). An ongoing clinical trial will compare 2-year overall survival and outcomes related to SCD in patients that undergo transplant compared with current standard of care (ClinicalTrail.gov Identifier: NCT02766465).

**TABLE 2:** Indications for HSCT balanced with donor availability: Risk/benefit ratio considerations.

Matched sibling donor	Matched unrelated donor or minimally mismatched good quality cord product	Mismatched marrow donor, haploidentical donor
• Stroke • Elevated TCD velocity • Acute chest syndrome • VOC • Pulmonary Hypertension/tricuspid regurgitation jet velocity.2.5 m/s • Osteonecrosis/AVN • Red cell alloimmunization • Silent stroke specially with cognitive impairment • Recurrent priapism • Sickle nephropathy	• Stroke • Elevated TCD velocity • Recurrent acute chest syndrome despite supportive care • Recurrent severe VOC despite supportive care • Red cell alloimmunization despite intervention plus established indication for chronic transfusion therapy • Pulmonary hypertension	• Recurrent stroke despite adequate chronic transfusion therapy • Inability to tolerate supportive care though strongly indicated, e.g., red cell alloimmunization, severe VOC and inability to take hydroxyurea

*HSCT, hematopoietic stem cell transplantation; AVN, avascular necrosis; TCD, transcranial doppler; VOC, vaso-occlusive crisis.*

In allogeneic transplant, the source of hematopoietic stem cells (HSCs) is from a donor (matched sibling, haplo-identical family members, UCB or MUD). Allogeneic BMT using HSCs from the latter 3 donor sources are still risky; and donor availability presents a huge limitation. These limitations can be overcome by autologous transplant, in which the patient receives his own cells after being modified by gene therapy.

#### Autologous Hematopoietic Stem Cell Transplant Modification: Gene Editing or Gene Therapy

Genetically engineered autologous cells eliminate the need to find a HSCT donor, and thus available to all patients. Since these are the patient’s own stem cells, there is no need for immunosuppression, thus eliminating the risks of GVHD and immune-mediated graft rejection (Esrick and Bauer, 2018; Orkin and Bauer, 2019).

Sickle cell disease patients represent a special and complicated population for this therapy for two major reasons. First, patients that undergo autologous stem cell transplant require collection of hematopoietic stem cells (CD34+) and the traditional method of collection is a bone marrow harvest done by a specialist but in patients with SCD this process yields CD34+ cells with suboptimal quantity and quality requiring multiple harvests, each harvesting procedure increasing the risk of triggering acute pain crisis. Second, the current gold standard procedure for cell mobilization is with granulocyte-colony stimulating factor (G-CSF) but this is contraindicated in patients with SCD due to risk of causing complications such as pain crisis, acute chest syndrome, and even death, from the increased white cell counts.

Recently, great advances have been made in using an alternative approach for harvesting CD34+ cells using Plerixafor. Plerixafor acts by reversibly blocking the binding between chemokine CXC-receptor 4 (CXCR4) and the stromal cell derived factor-1α triggering the mobilization of progenitor cells into the peripheral blood. It allows peripheral mobilization of stem cells by releasing CD34+ cells from the bone marrow niches, without the massive increase in white blood cells. Its development has been crucial in optimization of CD34+ collection in patients with SCD. Results have shown appropriate mobilization of CD34+ cells 6 h after a single dose of Plerixafor and are of higher quality and purity, decreasing the need for multiple bone marrow harvests and the associated stress/pain. Associated with hyper-transfusion therapy, it has become the preferred way of marrow stimulation to yield appropriate hematopoietic stem/progenitor cells in patients with SCD (Boulad et al., 2018; Esrick et al., 2018; Hsieh and Tisdale, 2018; Lagresle-Peyrou et al., 2018).

The genetic defect in the sickle HSPCs can be corrected via several approaches.

##### (A) Gene addition using lentiviral vector-based strategies

*(a) Anti- or non-sickling strategies:* Several gene therapies based on gene addition using viral vectors to carry therapeutic genes in HSCs are being actively developed with curative purposes. Gene addition strategies that have reached clinical trials include a promising one where the patient’s stem cells are infected with a lentivirus expressing an anti-sickling β-globin variant, T87Q. The unique feature of this vector is that the amino acid substitution (β^*A–T*87*Q*^) allows for high performance liquid chromatography (HPLC) monitoring of the transgene globin levels in the patient’s cells (Cavazzana-Calvo et al., 2010). The first SCD patient who received this Bluebird vector (protocol HGB-205) was reported in 2017; engraftment was stable with no sickle cell crises reported at 15 months of follow up (Ribeil et al., 2017), with further undergoing studies (ClinicalTrials.gov Identifier: NCT02140554, NCT03282656). Other approaches to anti-sickling gene therapy in erythroid-specific lentiviral vectors include utilizing a β-globin gene with three specific point mutations that confer anti-sickling properties (ClinicalTrials.gov Identifier: NCT02247843) or the introduction of a γ-globin coding sequence in a β-globin gene to increase HbF levels and decrease HbS (ClinicalTrials.gov Identifier: NCT02186418) (Cavazzana et al., 2017). Thus far, the most promising of these LV vectors is the one utilizing anti-sickling β-globin variant, T87Q.

*(b) Hb F induction:* The well-established efficacy of increasing HbF has motivated both pharmacological and genetic approaches to HbF induction.

A gene addition approach that is already in clinical trials (ClinicalTrials.gov Identifier: NCT03282656) utilizes a lentiviral mediated erythroid specific short hairpin RNA (shRNA) for BCL11A. This shRNA is modified to target the specific gene and downregulate its expression (Brendel et al., 2016). As of December 2018, three adults have been enrolled, utilizing plerixafor mobilized HSC, all three patients showed prompt neutrophil engraftment, and at 2 months follow up, the average HbF was 30% (ASH abstract #1023 – 2018 ASH conference). Other lentiviral therapies using zinc-finger nucleases (ZFN) directed against the γ-globin promoter have been proposed. This would force an interacting loop between the LCR and γ-globin which would reactivate γ-globin production, increasing HbF and decreasing HbS production at the same time. These lentiviral-based approaches still need preclinical *in vivo* studies to address safety and specificity before they can be considered in human patients (Breda et al., 2016; Orkin and Bauer, 2019).

Viral vectors, such as lentivirus, are a great tool for gene therapy but these results underscore the need to develop gene transfer protocols that ensure efficient and consistent delivery of the therapeutic globin gene cargo to HSC. Their major limitations include:

(1) Their immunogenicity which can create an inflammatory response in the donor which can lead to degeneration of the transducted tissue, (2) they can produce non-specific toxins, (3) due to the semi-random integration to the genome, there is a theoretical risk of insertional mutagenesis, (4) they have limitations of transgenic capacity size. An additional challenge in SCD is the ability to maintain a persistent myeloid donor chimerism of >20% to prevent return of SCD symptoms (Fitzhugh et al., 2017). Due to these limitations, long-term monitoring of patients to evaluate both safety and efficacy is necessary. Until now, over the last decade of clinical trials, no genotoxicity secondary to LV vectors has been reported but the main challenge has been to keep the myeloid donor chimerism above the 20% threshold (Nayerossadat et al., 2012).

##### (B) Gene editing

Gene-editing corrects a specific defective DNA in its native location. SCD with its simple single base change presents a very attractive prototype. Over the last couple of decades, there has been a spectacular growth of such strategies, setting the scene for developing therapies that could precisely genetically correct a single base mutation in patient with SCD. These strategies include ZFNs, transcription activator-like effector nucleases (TALENs) and the clustered regularly interspaced short palindromic repeat (CRISPR)-associated nuclease Cas9 approach which is the most advanced of the three. The CRISPR-Cas9 technology typically make a double-stranded break (DSB) in a particular genomic sequence directed to that site by a guide RNA. The most common method of DSB repair is non-homologous end joining, often resulting in gene disruption or knockout. This strategy is currently being tested in a clinical trial (ClinicalTrials.gov Identifier: NCT03745287) in which the patient’s own *BCL11A* gene (a major inhibitor of γ-globin gene expression) is disrupted to induce HbF expression. BCL11A also has roles in lymphoid and neurological development but gene-editing for SCD exploits the erythroid-specific enhancers in intron 2 of the gene (Bauer et al., 2013; Brendel et al., 2016). CRISPR-Cas9 technology is also being explored to mimic the rare, genetic variants that promote expression of the γ-globin genes as in hereditary persistence of fetal hemoglobin (Traxler et al., 2016; Wienert et al., 2018). Disrupting the putative binding sites for γ-globin repressors like BCL11A to induce HbF production will be an attractive therapeutic strategy for both β-thalassemic and SCD patients (Masuda et al., 2016; Liu et al., 2018; Martyn et al., 2018). The ultimate challenge, however, is to genetically correct the mutation, a single nucleotide change in the codon of the globin gene from GAG to GTG, by providing a homology template with the correct sequence at the sixth codon. Although this has been completed in preclinical studies, current techniques do not allow for specific transversion mutations like those required to cure SCD in humans (Dever et al., 2016; Orkin and Bauer, 2019). The enormous selective advantage of red blood cells with normal hemoglobin or anti-sickling hemoglobin predicts that genetic modification of a proportion of HSCs (estimated 10–20%) may suffice as a one-off treatment (Fitzhugh et al., 2017). Before gene therapy can become a reality, however, many hurdles need to be overcome; genetically manipulated HSCs need to be able to retain long-term repopulating potential; pre-transplant conditioning is toxic and needs to be modified to reduce the morbidity. A clinical trial exploring antibody-mediated non-chemotherapy conditioning is being evaluated in patients with severe combined immunodeficiency, in an attempt to reduce the exposure to chemotherapy and its toxicities is currently recruiting patients (ClincialTrials.gov Identifier: NCT02963064). Further understanding of this technology could represent a new option for patients with SCD.

Although different gene strategies have reached clinical trials showing promising results they remain in early phases of development and allogeneic HSCT remain the only curative treatment modality for SCD. For the majority of patients without a MSD, haploidentical HSCT with recent promising data of improved overall survival presents an alternative for curative therapy. Multiple gene therapy strategies utilizing patient’s own stem cells, are also being pursued, but this has the disadvantage of myeloablative conditioning (Leonard et al., 2020).

In addition to great advances in HSCT and gene therapy, new pharmacological anti-sickling approaches have developed.

### (2) Targeting Hemoglobin S Polymerization

Approaches targeting HbS polymerization presents a very attractive strategy as this “puts out the fire” rather than dealing with the sequelae of the sickling event (Eaton and Bunn, 2017). HbF has long been known to have a major beneficial effect in SCD – increased intracellular HbF not only dilutes the intracellular HbS concentration but inhibits sickling as the mixed hybrid tetramers do not partake in HbS polymerization. Hydroxyurea (HU) works via induction of fetal hemoglobin (HbF, α2γ2) synthesis, but hydroxyurea is only partially successful as the increase in HbF is uneven and not equally present in all the red blood cells (Ware, 2015). Nonetheless, use of HU therapy in SCD has expanded substantially in recent years. Follow on studies include demontration of its efficacy and safety in the pediatric population (BABY HUG) (Wang et al., 2011), the Transcranial doppler with Transfusion Changing to Hydroxyurea Study (TWiTCH) that showed HU was comparable to blood transfusions for primary stroke prevention (Ware et al., 2016) although the Stroke with Transfusion Changing to Hydroxyurea study (SWiTCH) concluded that HU is not comparable to blood transfusion in secondary stroke prevention (Ware et al., 2011).

More recently, two clinical studies have shown that HU is relatively safe in Sub Saharan Africa, a setting with high infectious disease and SCD burden. Hydroxyurea has been shown to not only decrease complications from SCD such as VOC, acute chest syndrome, frequency of transfusions, death and infections – including malaria but also to be a feasible approach in these under-resourced countries (Opoka et al., 2017; Tshilolo et al., 2019).

Despite having a significant impact in patients with SCD, there are still multiple unanswered questions regarding HU. Its mechanism of action has not been fully understood and its impact on HbF will decrease over time. Older patients become more sensitive to the dosage and they require frequent blood tests and readjustment of their dose. Regardless of the advances, there is no clear evidence of the long-term effect of hydroxyurea in preventing end organ damage (Nevitt et al., 2017; Luzzatto and Makani, 2019). There is also conflicting evidence of the effects of HU on male fertility (DeBaun, 2014). Chronic complications of SCD such as recurrent episodes of priapism, asymptomatic testicular infarctions and primary hypogonadism have been described as potential etiologies of low fertility in male SCD patients. Studies in transgenic SCD mice showed that SCD itself was associated with inhibition of spermatogenesis and primary hypogonadism but when compared to HU (25 mg/kg/day), testicular volume was lower in those mice with SCD exposed to HU, inferring lower spermatogenesis. Berthaut et al. (2008) measured the semen quality of 4 patients with SCA at baseline and 4 years after starting hydroxyurea. In three of four patients the spermatozoan concentration continued to drop while patients were taking the medication and did not return to baseline after discontinuing HU (Berthaut et al., 2008). Although the evidence is limited, full disclosure regarding implications on male fertility should be given to patients and families in order to make an informed decision before starting HU (Jones et al., 2009).

Other than HU, other pharmacological options to increase HbF are still experimental undergoing clinical trials. Molecular studies on γ-globin identified regulatory elements in the gene expression and subsequent HbF production. Such molecules; histone deacetylase (HDAC), DNA methyltransferase 1 (DNMT1), BCL11A and SOX6 modifying HbF expression have been explored as possible therapeutic options.

One of the proposed mechanisms for HU effect on HbF is stimulation of cyclic guanosine monophosphate (cGMP). Phosphodiesterase 9 (PDE9) is a specific enzyme in charge of degrading cGMP and is highly present in neutrophils and RBCs of patients with SCD. A novel, potent and selective PDE9 inhibitor (IMR-687) has been shown to increase levels of cGMP and HbF without signs of myelosuppression in cell lines of patients with SCD. An open-label extension to a previous phase 2a study is ongoing in adults with SCD (ClinicalTrials.gov Identifier: NCT04053803) (McArthur et al., 2019).

Panobinostat is a pan HDAC inhibitor currently being studied in adult patients with SCD as a phase 1 study (ClinicalTrials.gov Identifier: NCT01245179). *In vitro* analysis of human erythroid progenitor cells that underwent shRNA knockdown of HDAC1 or HDAC2 genes resulted in increased levels of γ-globin but without altering cellular proliferation of the cell cycle phase.

Associated with HU, HDAC gene inhibition produced a more pronounced increase of γ-globin and HbF (Esrick et al., 2015).

DNA Methyltransferase 1 is involved in the shutting down of γ-globin gene after birth and its subsequent production. DNA methylransferase inhibitor 5-azacytidine was one of the chemotherapeutic agents used to reactivate HbF but it was quickly abandoned due to its toxicity and carcinogenicity. Decitabine, an analog of 5-azacytidine, is also a potent DNMT1 inhibitor with a more favorable safety profile but decitabine is rapidly deaminated and inactivated by cytosine deaminase, if taken orally. To overcome this limitation, a clinical study combines decitabine and tetrahydrouridine (THU), a cytosine deaminase inhibitor, as a therapeutic strategy for inducing HbF (ClinicalTrials.gov Identifier: NCT01685515). In a phase 1 study, Molokie et al. (2017) showed that the inhibition of DNMT1 led to appropriate blood levels of decitabine that were safe and induced a large increase in fetal hemoglobin in healthy red blood cells. These agents did not induce cytoreduction, but increased platelets count that can potentially trigger vaso-occlusion in SCD patients (Molokie et al., 2017).

Voxelotor (Oxbryta/GBT440) binds specifically to the N-terminus of the alpha subunit of HbS to stabilize the oxygenated hemoglobin state (Strader et al., 2019), thus reducing the predisposition to sickling. Voxelotor (Oxbryta/GBT440) was approved by the FDA in November 2019 for the treatment of SCD in adults and pediatric patients 12 years of age and older. The HOPE study showed an increase in hemoglobin levels and reduced markers of hemolysis in 274 patients with HbS that were randomly assigned to receive the study drug versus placebo. These findings have not correlated with reduced episodes of pain crisis and/or end organ damage. Agents that shift Hb oxygen affinity present some concerns of potential negative effects as the bound oxygen cannot be off loaded in tissues with high oxygen requirements, particularly concerning in a disease characterized by decreased oxygen delivery (Hebbel and Hedlund, 2018; Thompson, 2019). These concerns are being addressed in a current phase 3, double-blind, randomized, placebo-controlled, multicenter study of Voxelotor (ClinicalTrials.gov Identifier: NCT03036813) (Vichinsky et al., 2019).

Dehydration of the RBC appears to be closely controlled by the efflux of potassium through 2 specific pathways; one is the potassium chloride cotransport and the other, calcium-activated potassium efflux (Gardos channel). Senicapoc blocks the Gardos channels, thus preventing dehydration of the red cells. Preclinical and phase 1/2 showed that inhibition of potassium flow through the Gardos channel increased Hb levels and decreased hemolysis (ClinicalTrials.gov Identifier: NCT00040677). A phase 3 study was terminated for lack of efficacy (ClinicalTrials.gov Identifier: NCT00294541) (Ataga et al., 2008; Ataga and Stocker, 2009).

*N*-Methyl D-aspartate receptors (NMDARs) are non-selective calcium channels present in erythroid precursors and circulating RBCs and have been shown to be abnormally increased in RBCs of patients with SCD (Hanggi et al., 2014). These channels are closely related with RBC hydration that affects the intracellular HbS concentration and thereby HbS polymerization and sickling of RBCs. Memantine is a NMDAR inhibitor which has shown to improve hydration of RBCs of patients with SCD *in vitro* and to reduce sickling in the setting of deoxygenation. It is being explored in an ongoing phase 2 clinical trial (ClinicalTrials.gov Identifier: NCT03247218).

Sanguinate which is a bovine PEGylated hemoglobin product attempts to block polymerization by targeting carbon monoxide (CO) delivery. By binding to HbS polymers, CO enhances their melting and minimize their persistence in peripheral blood. However, this equilibrium is based on high concentrations of CO. A phase 1/2 single-blind, randomized, placebo-controlled study of this agent in the management of pain crisis has been carried out but no results have yet been posted (ClinicalTrials.gov Identifier: NCT02411708).

### (3) Targeting Vasocclusion

Increased expression and activation of normally inactive erythroid adhesion molecules promote cytoadherence of sickle RBCs to the endothelium accompanied by platelets and leukocytes. Activated leukocytes and platelets further increase the risk to develop VOC (Nasimuzzaman and Malik, 2019; Sundd et al., 2019; Telen et al., 2019).

Previous *in vitro* studies had demonstrated that glutamine depletion contributed to red blood cell membrane damage and adhesion. Uptake of L-glutamine uptake is markedly increased in patients with SCD, primarily to increase the total intracellular NAD level (Morris et al., 2008). In a phase 3 study, L-glutamine demonstrated a 25% reduction in the median number of pain crisis, 30% less hospitalizations and reduced acute chest episodes in children and adults with SCD with or without HU over a 48-week period. There were 36% drop-out rate in the glutamine arm and 24% in the placebo control arm from unknown reasons. L-Glutamine appears to significantly increase NADH and NAD redox potential and decrease endothelial adhesion, but its mechanism remains still unknown and there are concerns regarding its use in patients with renal impairment, a common sickle-related complication (Quinn, 2018). In July 2017, the pharmacological grade of L-glutamine (Endari) was approved by the FDA for use in patients with SCD, 5 years or older (Niihara et al., 2018). Of note, L-glutamine has not been approved by the European Medicines Agency for treating SCD.

In the future it could be a useful combination therapy with HU (Minniti, 2018) but uptake among patients is still low, one of the reasons is the unpleasant taste. There are potentially less expensive pharmaceutical formulations of L-glutamine available off the counter, but purity of the effective agents in these compounds have not been validated.

As the endothelium emerge as a key factor in the constant activation of adhesion molecules in sickle RBCs, these adhesion molecules present a very attractive therapeutic target. Selectins, which are present in endothelial cells and are the initial step toward a firm adhesion between RBCs and the endothelium, have been further studied and targeted as possible therapeutic approaches.

Crizanlizumab is a monoclonal antibody to P-selectin and its mechanism of action is to block the adhesion of activated erythrocytes, neutrophils and platelets. In a phase 2, multicenter, randomized, placebo controlled double blind study, crizanlizumab with or without hydroxyurea (SUSTAIN study) (ClinicalTrails.gov Identifier: NCT01895361) showed that patients on the treatment arm had significantly lower rate of sickle-related pain crises compared to placebo with a lower incidence of adverse events - 10% of patients suffered from moderate side effects while one patient suffered from an intracranial bleed during treatment with this drug that could also interfere with platelet function via its effects on selectins (Ataga et al., 2017). *Post hoc* analyses showed that more patients were VOC event-free in the crizanlizumab arm than in the placebo arm, and that crizanlizumab also significantly increased time-to-first VOC compared to the placebo (Kutlar et al., 2019). A phase 3 interventional, multicenter, randomized, double-blind clinical trial is ongoing to assess safety and efficacy of crinalizumab with or without hydroxyurea in patients with SCD and history of VOC (ClinicalTrials.gov Identifier: NCT03814716). In November 2019, the US Food and Drug Administration approved crizanlizumab-tmca (ADAKVEO, Novartis) to reduce the frequency of VOC in adults and pediatric patients aged 16 years and older with SCD.

Rivipansel is a pan-selectin inhibitor with its strongest activity against E-selectin. In a multicenter, randomized, double−blind, placebo−controlled phase 2 study (ClinicalTrails.gov Identifier: NCT01119833), Rivipansel showed clinical and meaningful reductions in multiple measures of VOC compared with those receiving standard of care treatment (Telen et al., 2015). A phase 3 study (Identifier: NCT02187003) to evaluate the efficacy and safety of rivipansel in the treatment of VOC in hospitalized patients with SCD was terminated (posted on ClinicalTrials.gov February 20, 2020) based on failure of the primary study (NCT02433158) to meet the study efficacy endpoints of time to readiness-for-discharge.

In a phase 1, dose-escalation study propranolol showed it significantly reduced epinephrine-stimulated sickle RBCs adhesion. A phase 2 study (NCT01077921) showed decrease in adhesion molecules such as E-selectin and P-selectin but results were not statistically significant and no clinical endpoints were discussed (De Castro et al., 2012).

Due to their P-selectin mediated adhesion inhibition properties, heparinoids have been additionally investigated with interesting results. Sevuparin, a heparin derivate polysaccharide that has shown to bind to P− and L−selectins, thrombospondin, fibronectin and von Willebrand factor, all of which are thought to contribute to vasocclusion in SCD. It has been reported to inhibit sickle RBC adhesion to the endothelial cells and to reduce tumor necrosis factor-induced vasocclusion. It is currently being tested in a phase 2 clinical trial, placebo controlled, to study its efficacy and safety in patients with SCD during VOC (ClinicalTrials.gov Identifier: NCT02515838) (Telen et al., 2016). Other heparinoids such as Dalteparin showed incomplete evidence to support or refute its effectiveness in the management of patients with SCD. There are ongoing trials (ClinicalTrials.gov Identifier: NCT02098993) to assess the feasibility of unfractionated heparin in patients with SCD admitted with pain crisis. Well-designed studies are still needed to clarify its role in the management of patients with SCD and to assess the safety of this approach (van Zuuren and Fedorowicz, 2015).

Poloxamer 188 is a non-ionic block copolymer surfactant thought to seal stable defects in the microvasculature leading to an improvement in blood flow and decreasing blood viscosity. Although its mechanism is not well understood, a randomized, double-blind, placebo-controlled trial showed that it decreased the duration of sickle crisis by 8 h compared to placebo (133 h vs. 141 h, *p* = 0.04) and more patients receiving the medication reported crisis resolution (52% vs. 37%, *p* = 0.02) (Orringer et al., 2001). In an early phase 2 study, one patient receiving the medication developed renal dysfunction due to presence of low molecular weight substances and a purified version was designed (Adams-Graves et al., 1997). Vepoloxamer, a purified form of Poloxamer 188 with multi mechanistic properties, was believed to improve RBC adhesion, membrane fragility and organ damage. Unfortunately, a phase 3 study failed to reduce the mean duration of VOC in patients with SCD compared to placebo (Adams-Graves et al., 1997).

### (4) Targeting Inflammation

Continual background inflammation contributes to organ damage in patients with SCD. Persistent activation of platelets, neutrophils, monocytes, endothelium, and coagulation factors are key participants in this vicious cycle. Different therapeutic approaches have been proposed to assess the impact in patients with SCD (Nasimuzzaman and Malik, 2019; Sundd et al., 2019; Telen et al., 2019).

Intravenous immunoglobulin (IVIG) and statins have been studied for their anti-inflammatory effects on neutrophils and monocyte adhesion. Patients on statin demonstrated a decrease in C-reactive protein, soluble ICAM1, soluble E-selectin and vascular endothelial growth. Simvastatin was found to reduce adhesion of white blood cells and in combination with hydroxyurea, was found to decrease the number of pain crisis and markers of inflammation (Hoppe et al., 2017). Currently, there is an active clinical trial to assess the effect of simvastatin on central nervous system vasculature in patients with SCD (ClinicalTrials.gov Identifier: NCT03599609).

*N*-Acetylcysteine (NAC) commonly used in respiratory conditions has also been tested for patients with SCD. In a phase 2 study, NAC proved to inhibit dense cell formation and restored glutathione levels toward normal. The decrease in irreversible sickling of RBCs was not statistically significant but a downward trend was observed (Pace et al., 2003; Nur et al., 2012). Further studies have shown decreased red cell membrane expression of phosphatidylserine which seems to reflect overall reduced oxidative stress. To better assess its clinical effect in patients with SCD, a pilot study, currently enrolling with invitation is studying its effect in redox and RBC function during VOC (ClinicalTrials.gov Identifier: NCT01800526).

Aberrant activation of the coagulation cascade, abnormal excess of TF on the endothelial wall and high plasma levels of different coagulation factors drive increased thrombin and fibrin production leading to further inflammation and risk of VOC (Sundd et al., 2019). In a SCD mouse model, factor Xa, TF, and thrombin differentially contributed to vascular inflammation (Sparkenbaugh and Pawlinski, 2013). Factor Xa inhibition demonstrated a decrease in vascular inflammation as assessed by the lower interleukin 6 levels. Although thrombin had no effect on interleukin 6, it was a significant factor for neutrophil infiltration and further inflammation (Sparkenbaugh et al., 2014). A retrospective analysis of rivaroxaban, a factor Xa inhibitor, demonstrated non-inferiority with regard to thrombosis compared to warfarin with the advantage of less outpatient visits and monitoring (Bhat and Han, 2017). Currently, a two-treatment phase clinical trial with rivaroxaban on the pathology of SCD has been completed but results are pending (ClinicalTrials.gov Identifier: NCT02072668). Patients with SCD have increased platelet levels at baseline that are further increased during acute VOC. Platelet activation triggers further leukocyte activation and promote RBC adhesion to an exposed endothelium (Conran and Belcher, 2018) setting off a vicious cycle of adhesion events. Antiplatelet therapy with Clopidogrel in patients with SCD, unfortunately, were disappointing. New, third generation P2Y12 inhibitors such as ticagrelor and prasugrel have also been studied in patients with SCD. Prasugrel showed appropriate levels of anti-platelet aggregation compared to healthy patients in *ex vivo* studies, and was well tolerated by patients, but on a 24-month follow up, patients on the treatment arm failed to show reduction in the frequency of VOC (Heeney et al., 2016; Conran and Rees, 2017). Ticagrelor, in a phase 2b study, was well tolerated, but failed to show effect in the frequency of VOC (Kanter et al., 2019) (ClinicalTrials.gov identifier: NCT02482298). Previous studies have also showed that aspirin as an anticoagulant therapy did not provide benefit over placebo, although it is used as an analgesic in many parts of Africa (Sins et al., 2017).

In patients with SCD, continual lysis of RBCs activates the inflammasome triggering the release of multiple cytokines, including IL-1β (Awojoodu et al., 2014). Canakinumab is a humanized monoclonal antibody that targets interleukin 1-β (IL-1β), and thus potentially could be useful in mitigating some of the inflammation in SCD. Canakinumab was shown to be well tolerated and not associated with major side effects in pediatric and young adult patients (Rees, 2019). A clinical trial to assess its efficacy, safety and tolerability is ongoing in the pediatric population (ClinicalTrials.gov Identifier: NCT02961218).

## Conclusion

**FIGURE 3:**
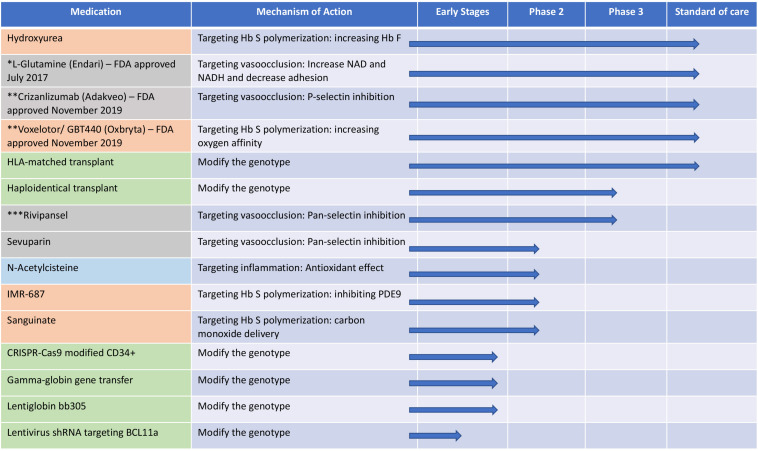
The different therapeutic approaches for sickle cell disease and their mechanisms and current status in clinical trials. Orange: targeting hemoglobin S polymerization; gray: targeting vasocclusion; light blue: targeting inflammation and green: modification of the genotype. shRNA, short hairpin RNA; Hb S, hemoglobin S; Hb F, hemoglobin F; PDE9, phosphodiesterase 9. *FDA approved July 2017; **FDA approved November 2019; ***Terminated in February 20, 2020 due to failure to meet primary endpoints.

In the last 30 years, there has been a revolution in the medical sciences, and SCD because of its genetic simplicity, has been at the forefront of the numerous scientific discoveries. Tremendous progress has been made in understanding its pathophysiology and pathobiological complexities, but developing treatments, has been disproportionately slow and elusive. However, after a century of neglect, going back to basics offers hope for translating these insights into better therapeutic options – pharmacological and genetic – and for finding curative genetic options for SCD (Figure 3). Although frequent in the US, SCD is far more prevalent in Africa where patients have less access to resources, medical treatment and facilities and the consequences of the disease are devastating. As we move forward, we have to continue focus our therapeutic approaches so that they can be accessed by those that suffer the most.

## Author Contributions

GSC and ST wrote and revised the manuscript.

## Conflict of Interest

The authors declare that the research was conducted in the absence of any commercial or financial relationships that could be construed as a potential conflict of interest.

## Footnotes

1 https://apps.who.int/iris/handle/10665/20890
